# Multivesicular bodies: co-ordinated progression to maturity

**DOI:** 10.1016/j.ceb.2008.04.001

**Published:** 2008-08

**Authors:** Philip G Woodman, Clare E Futter

**Affiliations:** 1 Faculty of Life Sciences, University of Manchester, Oxford Road, Manchester M13 9PT, United Kingdom; 2 UCL Institute of Ophthalmology, 11-43 Bath Street, London EC1V 9EL, United Kingdom

## Abstract

Multivesicular endosomes/bodies (MVBs) sort endocytosed proteins to different destinations. Many lysosomally directed membrane proteins are sorted onto intralumenal vesicles, whilst recycling proteins remain on the perimeter membrane from where they are removed via tubular extensions. MVBs move to the cell centre during this maturation process and, when all recycling proteins have been removed, fuse with lysosomes. Recent advances have identified endosomal-sorting complex required for transport (ESCRT)-dependent and ESCRT-independent pathways in intralumenal vesicle formation and mechanisms for sorting recycling cargo into tubules. Cytoskeletal motors, through interactions with these machineries and by regulating MVB movement, help to co-ordinate events leading to a mature, fusion-competent MVB.

## Introduction

**Figure 1:**
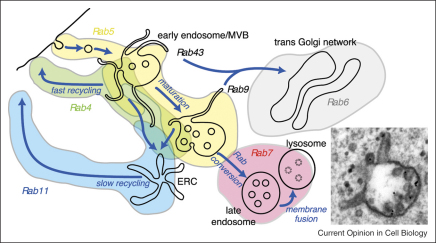
An overview of endosomal maturation. The early endosome/immature multivesicular endosome/body (MVB) consists of vacuoles and connecting tubules that carry recycling cargo. Overlapping pools of Rab GTPases confer functional and morphological properties to each domain (assignment of Rab proteins involved in early endosome to TGN transport is based on [62]). During maturation the vacuoles enlarge and increase their complement of internal vesicles whilst the amount of tubules diminishes. The final act during maturation is Rab conversion, at which point the endosome loses the ability to exchange material and becomes competent to fuse with the lysosome. The inset shows an example of an MVB 15 min after internalisation of EGF. The gold particles are conjugated to anti-EGFR.

The early endosome is a pleiomorphic structure composed of vacuolar and tubular domains that exhibit extensive connectivity but maintain their identity by virtue of their specific complement of Rab GTPases (see Figure 1). With time, cargo destined for degradation concentrates on intralumenal vesicles that accumulate within the vacuolar domains, giving rise to multivesicular endosomes/bodies (MVBs), whilst recycling proteins are removed via tubular domains. Accompanying the maturation process, the MVB moves from the cell periphery to the cell centre and, when all the recycling proteins have been removed, interacts, either via direct fusion [1] or via a kiss and run mechanism [2], with a stable compartment that we term the lysosome. This latter compartment contains lysobisphosphatidic acid (LBPA) and LAMPs and has sometimes been termed the late endosome. However, we believe the lysosome is a more appropriate term, as it is where endocytosed ligands and receptors are degraded, and the late endosome is a term more appropriately used for the mature MVB that has lost all recycling proteins and is competent to fuse with the lysosome. The final progression of the MVB to this fusion-competent state is marked by Rab5–Rab7 conversion [3] and the resulting switch in the repertoire of Rab effector proteins on the endosome membrane.

Given the iterative nature of receptor recycling coupled with the high fidelity of sorting achieved, it is not surprising that MVB maturation can be extended (30 min or more in EGF-stimulated cells [1,3,4]); perhaps more surprising is the short lifetime of the fully mature MVB, such that in more than one study this compartment could not be kinetically resolved without experimental manipulation [1,3]. This implies that the processes of cargo sorting, movement to the cell centre and acquisition of fusion competence are co-ordinated.

Here we review recent data pointing towards mechanisms underlying intralumenal vesicle formation and the generation of recycling tubules and discuss how molecular connections between these events and membrane movement could contribute to the co-ordination of MVB maturation.

## Intralumenal vesicle formation: the role of ESCRTs

Lysosomal targeting of endocytosed membrane proteins requires specific signals, of which the best characterised is ubiquitination. Ubiquitinated cargo engages a series of proteins/protein complexes, first identified from studies of vacuolar protein sorting (*VPS*) mutants in yeast and collectively termed Class E VPS proteins. These proteins package cargo onto intralumenal vesicles of MVBs. Most Class E VPS proteins are found within the endosomal-sorting complex required for transport (ESCRT) 0–III, and the role of these complexes in sorting ubiquitinated cargo has recently been reviewed elsewhere [5,6]. That the ESCRT machinery also has a role in intralumenal vesicle formation is suggested by the demonstration that ESCRT depletion/deletion inhibits the formation of intralumenal vesicles [7–9]. This may, in part, be an indirect effect of inhibiting sorting of components of the inward vesiculation machinery or cargo. Activated EGF receptor (EGFR), a well-characterised mammalian MVB cargo, itself leads to an increase in the production of intralumenal vesicles [10^•^] and the effects of ESCRT depletion on endosome morphology are more profound in EGF-stimulated than in unstimulated cells [7].

The ESCRT complex most likely to be involved directly in intralumenal vesicle formation is ESCRTIII, which does not itself bind ubiquitin. Recent structural data suggest possible models whereby ESCRTIII components (also known as charged multivesicular body proteins or CHMPs) could achieve this. CHMPs are likely to multimerise to form a flat lattice on the perimeter membrane of the endosome [11,12^•^]. Such lattices could either modify the lipid composition of the underlying membrane to induce budding or bridge the gap over an invaginating bud to promote fission, or delineate the area where inward budding takes place. ESCRT complexes dissociate from the perimeter membrane of endosomes before inward vesiculation occurs through the activity of the ATPase, Vps4. Controlled removal of individual CHMPs could reduce the size of the lattice, reducing the distance between membranes of opposing sides of the bud and thus promoting fission. Overexpressed CHMP4A and 4B can form curved filaments on the cytoplasmic face of the plasma membrane that, in the presence of dominant negative Vps4, promote negative membrane curvature [13^•^]. Thus, these ESCRT components are capable of promoting membrane curvature in the direction required for inward invagination.

However, despite the range of structural data, evidence for a direct role for ESCRTIII in intralumenal vesicle formation remains elusive. In mammalian cells, for example, although depletion of a component of ESCRTIII, VPS24/CHMP3, reduced the number of internal vesicles per MVB, EGFRs were still sorted onto the smaller number of intralumenal vesicles that formed [14]. This implies that at least this ESCRTIII subunit may be dispensable for some types of inward vesiculation. Of course, since the ESCRTIII complex in mammalian cells has six CHMPs, some with multiple isoforms, one explanation for this finding is that CHMPs may be partially redundant. Vps4 deletion in yeast [9] and expression of dominant negative Vps4 in mammalian cells [15] inhibit intralumenal vesicle formation, consistent with removal of ESCRTIII subunits being important for this process. However, deletion of Did2 in yeast, which is required for Vps4-mediated dissociation of ESCRTIII but not ESCRTI or II, does not prevent intralumenal vesicle formation [9].

## Other mechanisms for intralumenal vesicle formation

Although ESCRTs may well play a role in intralumenal vesicle formation, accumulating data suggest that, at least in mammalian cells, other mechanisms/machineries also participate in this process and that an interaction of the protein machinery with the lipids of the underlying membrane is crucial to both ESCRT-dependent and ESCRT-independent mechanisms.

LBPA is a cone-shaped lipid that has been implicated in either inward vesiculation or back-fusion of internal vesicles with the limiting membrane in a manner promoted by Alix, which binds to LBPA-containing liposomes [16,17]. Alix provides a link with the ESCRT machinery, as it binds both ESCRTI and ESCRTIII [18]. However, LBPA has not been found in yeast and is present in lysosomes and a subset of MVBs that are distinct from those that traffic EGFR [10^•^], and so is unlikely to form part of the core inward vesiculation machinery. Another lipid that may be important for MVB formation is PI3,5P_2_. ESCRTIII binds PI3,5P_2_ [19], which may allow it to localise to MVBs independently of ESCRTs I and II. The PI3P 5-kinase, Fab1p, has been implicated in sorting of cargo onto intraluminal vesicles in yeast [20]. Perturbation of the Fab1p orthologue, PIKfyve, in mammalian cells causes the generation of enlarged vacuoles [21,22], but MVBs with many internal vesicles are present in Drosophila PIKfyve mutants [23], suggesting PIKfyve may be dispensable for at least some types of inward vesiculation. That multiple mechanisms may exist for the formation of intralumenal vesicles is emphasised by a recent study showing that intralumenal vesicles destined for release from the cell surface in the form of exosomes form independently of ESCRTI and Vps4 and require the sphingolipid, ceramide [24]. This cone-shaped lipid may spontaneously generate the negative curvature required for intralumenal vesicle formation.

EGF-stimulated intralumenal vesicle formation requires the calcium-binding and phospholipid-binding protein, annexin 1 [10^•^]. Annexin 1 is not required for inward vesiculation in unstimulated cells. However, it is a substrate of the EGFR kinase and the single tyrosine phosphorylation site in the annexin 1 N-terminus is required for EGF-stimulated intralumenal vesicle formation. No link between annexin 1 and the ESCRT machinery is known and EGFR is transported to the limiting membrane of lysosomes in cells which lack annexin 1 [10^•^], suggesting that this protein is likely to have a direct role in intralumenal vesicle formation rather than cargo sorting. Annexin 1 can mediate vesicle aggregation *in vitro* and so one possible mode of action is for it to bring opposing membranes of the invaginating intralumenal bud together to promote fission [25].

Further evidence of ESCRT-independent pathways of intralumenal vesicle formation has come from studying the protein Pmel17, a main component of the c fibrils of premelanosomes, which is targeted to intralumenal vesicles of MVBs independently of ubiquitination, ESCRT0 and ESCRTI [26]. However, it remains possible that ESCRTIII could play a role in this form of intralumenal vesicle formation independently of ESCRTI.

## Membrane retrieval pathways

**Figure 2:**
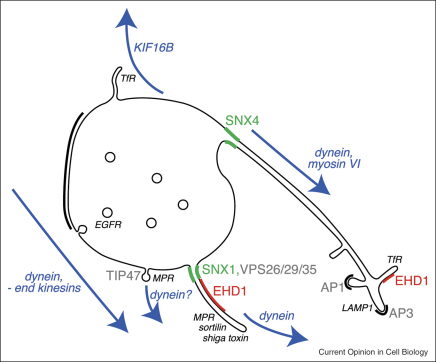
Retrieval pathways and movement. As the early endosome moves towards the cell centre, cargo (black italics) is retrieved using tubular or vesicular intermediates which themselves move using molecular motors. For simplicity, SNX1 and SNX4 are shown bound to the necks of emerging tubules, but may be enriched along the length of the tubule. The position of EHD1 relative to SNX4 during TfR recycling is not known. Clathrin coats (including buds along tubules and the flat clathrin lattice that localises to the vacuole and contains Hrs) are drawn in black. Cargo-specific adaptor proteins (grey) are also shown. Endosome motility is also controlled by the actin cytoskeleton (not shown).

As EGFR is concentrated into intralumenal vesicles, membrane cargoes destined for a range of other compartments are filtered away into tubular elements (reviewed in [27]). ‘Geometric sorting’, facilitated by the different membrane area/volume ratio of vacuolar and tubular domains, may account to some extent for this separation. However, for at least some cargoes a picture is emerging how sorting could be coupled directly to membrane tubulation (Figure 2).

Mannose-6-phosphate receptors (MPRs) deliver soluble hydrolases to the endosomal system. After ligand dissociation, the vacant receptors return to the TGN. Retrieval may occur at different points during endosomal maturation, with each pathway using a distinct sorting apparatus. One population of MPR is retrieved late, consistent with the localisation of a portion of MPR to mature MVB [28^•^]. Routing of MPR via this pathway requires Rab9, as well as TIP47 [29]. Other studies have found that the retromer complex of SNX1, SNX2, VPS26, VPS29 and VPS35 is essential for efficient retrieval of MPR [30–32] and the functionally related receptor sortilin [28^•^], and for endosome–TGN transport of Shiga toxin, from an earlier point during endosome maturation [33]. Retrieval of MPR and trafficking of Shiga toxin also requires clathrin-specific and cargo-specific adaptor proteins (see [27] for review), though the functional relationship between these and Rab9/TIP47-based or retromer-based transport is not fully resolved (see [27,33]).

MPR and sortilin localise to SNX1-enriched tubules (termed endosome–TGN transport carriers or ETCs) that emanate from relatively early MVBs. Indeed, retromer links cargo selection with tubule generation, since the VPS26/29/35 retromer subcomplex binds cargo [34] and clusters it within the endosomal membrane [28^•^] whilst the linear structure of this subcomplex could potentially accommodate highly curved membrane [34]. SNX1 (and perhaps SNX2) may actually drive or at least stabilise membrane tubulation by virtue of a phox homology (PX) domain that recognises the endosomal membrane lipid PI-3-P, and a neighbouring Bin/Amphiphysin/Rvs (BAR) domain that can either sense or induce membrane curvature [30]. Hence, multimers of retromer could enclose cargo within membrane tubules [34]. These tubules could be stabilised by EHD1, a member of the C-terminal Eps15-homology (EH)-domain protein family widely involved in endocytic trafficking [35]. EHD1 binds to retromer and depletion of EHD1 reduces the number and linearity of SNX1-containing tubules and delays recycling of MPR [36^•^]. The closely related protein, EHD2, is an ATPase that induces tubulation of artificial liposomes *in vitro* [37^••^].

Transferrin receptor (TfR) is recycled from the early endosome back to the cell surface, either directly, or more slowly via an endosomal-recycling compartment (ERC). TfR is found within a convoluted tubular endosomal network (TEN) [38], which can be distinguished morphologically from ETCs arising from the same early endosomal vacuoles [28^•^] and which presumably represents an intermediate on the TfR recycling pathway. TENs may act as platforms for the retrieval of a range of endosomal cargoes, since they also contain buds labelled for clathrin/AP-3, which are responsible for trafficking LAMP-1 directly between the early endosome and the lysosome limiting membrane, as well as separate buds labelled for clathrin/AP-1 [38]. Whether entry into the TEN itself is selective is not yet clear. However, a clue may come from the recent discovery that SNX4, which contains both PX and BAR domains, is required for TfR recycling [39^••^]. In live cells, GFP-SNX4 is localised with TfR in tubules that emanate from early endosomes and which are distinct from those containing mCherry-SNX1, consistent with the existence of morphologically distinct transport intermediates arising from the same endosome [28^•^]. Depletion of SNX4 results in a loss of TfR, but not MPR, to the degradative pathway [39^••^], whilst loss of SNX1 results in the reverse [30]. These data might argue that entry to both recycling pathways is selective. Interestingly, TfR recycling also involves EHD1 [40], though the relationship between EHD1 and SNX4 has not been explored.

## Cargo sorting and cytoskeletal motors

One emerging area of interest is the role of cytoskeletal motors in separating recycling receptors away from degradative cargo and delivering them to their target organelles. The early endosome moves towards the cell centre as it matures. This movement is mediated by cytoplasmic dynein (dynein [4]), a minus end-directed microtubule motor, as well as a minus end-directed kinesin-like activity controlled by Rab5 [41]. Several target compartments (ERC, TGN, lysosomes) are also localised close to the cell centre and transport to these is likely to depend on dynein or other minus end-directed microtubule-based and/or actin-based motors. Indeed, dynein has been implicated directly in tubule-mediated trafficking between the early endosome and ERC by the finding that SNX4 binds to KIBRA, which interacts with dynein light chain 1 [39^••^]. The retrograde myosin, myosin VI is also involved in this pathway [42]. Rab9-containing carriers have been visualised moving bidirectionally, probably on microtubules [43], and dynein is involved in the transport of Shiga toxin from the endosome to the TGN [44^•^]. Cytoskeletal motors also participate in the direct recycling of TfR from the early endosome. The kinesin KIF16B is recruited to early endosomes via a PX domain and supports recycling [45]. KIF16B could achieve this either by enhancing the formation/transport of microtubule plus end-directed carriers or by maintaining the vacuolar early endosome in the periphery for a period by opposing minus end-directed motor activities.

An important question yet to be addressed is whether cytoskeletal motors merely translocate pre-formed tubular carriers towards their target, or contribute to the formation, extension or scission of recycling tubules. Certainly, SNX1 tubules require microtubules for their stability [36^•^]. In addition, under at least some circumstances dynein can aid the formation of membrane tubules, since ER tubules extend rapidly along microtubules *in vitro* when dynein is present [46]. The tension generated as the microtubule motor applies force to tubules that are immobilised at one end causes the tubules to deform [46] and this situation within a recycling tubule could conceivably favour the selection of specific lipids and membrane cargo and so further contribute to sorting. Although ER tubules placed under tension in this way seldom break [46], scission of artificial lipid tubules can be induced experimentally by combining microtubule motor-induced tension with dynamin, a protein that promotes nucleotide hydrolysis-dependent membrane scission at the plasma membrane *in vivo* [47^••^]. EHD proteins, or unknown factors, may act in an analogous fashion at the endosome [37^••^]. However, the maturing MVB is not immobilised; it moves towards the cell centre, in the same direction as recycling tubules whose target compartment is the ERC or TGN. How can cytoskeletal motors promote carrier formation under these circumstances? One clue might be found in observations that endosomes move in a highly saltatory manner, with short bursts of inward movement being interspersed with phases in which they are relatively immotile or actually move outwards [4], presumably using KIF16B. It may be during these phases that separation of recycling cargo is most pronounced. Several candidate molecules could modulate the motility of endosomes by attaching them to the actin cytoskeleton. For example, HAP40 mediates the switching of endosomes from microtubules to actin filaments, and overexpression of HAP40 dramatically reduces endosome motility [48^•^]. In addition, Rho D and Rho B regulate actin assembly around early endosomes via the action of Diaphanous-related formins [49,50] and myosin 1b regulates the position and lumenal content of MVB [51].

## Acquisition of maturity

MVB maturity is marked by the complete removal of recycling proteins and the acquisition of the ability to fuse with the lysosome. How are the two co-ordinated? One way would be to ensure that MVBs and lysosomes do not come into close proximity until recycling proteins have been removed. Dynein not only regulates removal of TfR from the maturing MVB and movement of the MVB to the cell centre [4] but also may regulate lysosome movement through interaction, via dynactin, with the Rab7 effector, RILP [52,53]. It could therefore couple recycling to the bringing together of MVBs and lysosomes. However, additional mechanisms probably operate, because dynein may not be the major motor regulating lysosome movement [54] and maturing MVBs can move to the cell centre before the complete removal of recycling proteins and before Rab5–Rab7 conversion [3,4].

Proteins on the MVB required for MVB–lysosome fusion include Rab7, the tethering proteins, which comprise the HOPs and CORVET complexes, and a specific set of SNAREs (reviewed in [55]). The HOPs complex is required for Rab5–Rab7 conversion on maturing MVBs [3]. The fusion-competent mature MVB may have lost ESCRT complexes, as sequestration of cargo in intralumenal vesicles of MVB is accompanied by ESCRT complex dissociation. Could ESCRT activity or disassembly be coupled to acquisition of fusion competence? The interaction between ESCRTII and RILP [56,57] provides a potential link between ESCRTs and the Rab7/HOPs machinery. Indeed, RILP depletion inhibits intralumenal vesicle formation [58]. Deubiquitinating enzymes (DUBs) are also candidates for coupling ESCRT disassembly to acquisition of fusion competence. These enzymes interact with the ESCRT machinery and remove ubiquitin from cargo before inward vesiculation, but may have multiple targets in addition to cargo, including the ESCRT machinery itself and the MVB–lysosome fusion machinery [59]. These ESCRT interactions potentially couple sorting of degradative cargo to acquisition of fusion competence. Components of the ESCRT complexes have also been implicated, either directly or indirectly, in certain receptor recycling pathways [8,60,61], but further studies are required to determine whether these interactions might also couple completion of recycling to fusion competence.

## Conclusions

The past few years have seen a great advance in our understanding of the molecular requirements for the events leading to MVB maturation. Components needed for inward and outward budding of the MVB perimeter membrane to generate intralumenal vesicles and recycling tubules, respectively, have begun to be identified. However, the mechanics of these processes are not yet clear and the requirements for scission of intralumenal vesicles and recycling tubules remain unknown. Some of the machinery required for cargo selection, intralumenal vesicle formation, recycling tubule formation and MVB–lysosome fusion interact with each other, with Rab proteins and with components of the cytoskeleton. Determining how these processes are coupled mechanistically and hence co-ordinate MVB maturation is a major future challenge.

## References and recommended reading

Papers of particular interest, published within the period of review, have been highlighted as:

• of special interest
•• of outstanding interest
